# Hemoperfusion with HA380 in acute type A aortic dissection patients undergoing aortic arch operation (HPAO): a randomized, controlled, double-blind clinical trial

**DOI:** 10.1186/s13063-020-04858-2

**Published:** 2020-11-23

**Authors:** Jing Yang, Dong Ji, Yue-Qian Zhu, Yun Ren, Xun Zhang, Hong-Yu Dai, Xu Sun, Yi Zhou, Zhi-Yuan Chen, Qing-Guo Li, Hao Yao

**Affiliations:** Cardiovascular Center, The Second Affiliated Hospital of Nanjing Medical University, Nanjing, 210011 Jiangsu Province China

**Keywords:** HA380, Acute type A aortic dissection, Cardiopulmonary bypass, Deep hypothermic circulatory arrest, Inflammatory factors, Coagulation indicators

## Abstract

**Background:**

Cardiopulmonary bypass (CPB) is an important cause of significant systemic inflammatory response syndrome (SIRS) in the surgical treatment of acute type A aortic dissection (ATAAD). In patients with arch vessel involvement, extensive surgical repairs often necessitate prolonged use of CPB and results in extensive inflammatory responses. Cytokines and chemokines released during CPB contribute to the progression of SIRS, increase perioperative complications, and negatively impact surgical outcomes. A cytokine adsorber (HA380) is expected to reduce the level of cytokines during CPB, which may decrease both intraoperative and postoperative inflammation. The purpose of this study is to investigate if HA380 is able to reduce the levels of inflammatory cytokines and decrease perioperative complications in ATAAD patients undergoing CPB and deep hypothermic circulatory arrest (DHCA).

**Methods:**

This study is a single-center, randomized, controlled, double-blind clinical trial. The study aims to recruit 88 patients with ATAAD and aortic arch involvement who will undergo CPB and DHCA to repair the dissected aorta. Patients will be randomized equally into the CPB/DHCA only group (control group) and the CPB/DHCA + HA380 hemoperfusion group (intervention group), with 44 patients each. Patients in the control group will undergo CPB and DHCA only, while patients in the intervention group will undergo continuous hemoperfusion with HA380, in addition to CPB and DHCA. The primary outcome is a composite of major perioperative complications. The secondary outcomes include related inflammatory markers, coagulation parameters, and minor perioperative complications. To comprehensively evaluate the effect of hemoperfusion on the perioperative outcomes, we will also determine if there are differences in perioperative all-cause mortality, length of ICU stay, and total hospitalization costs.

**Discussion:**

In the current trial, hemoperfusion will be applied in patients undergoing CPB and DHCA for repair of the aorta involving the aortic arch. This trial aims to test the safety and efficacy of our hemoperfusion device (HA380) in such settings. Upon completion of the trial, we will determine if HA380 is effective in reducing perioperative proinflammatory cytokine levels. Further, we will also verify if reduction in the proinflammatory cytokine levels, if present, translates to improvement in patient outcomes.

**Trial registration:**

ClinicalTrials.gov NCT04007484. Registered on 1 July 2019 (retrospectively registered).

## Background

Cardiovascular disease is the leading cause of death worldwide [1]. In the last couple of decades, the advancement of cardiac surgery has enabled invasive management and improved overall outcomes in patients with heart and great vessel diseases. Cardiopulmonary bypass (CPB) is an indispensable technique used in open-heart surgery that ensures perfusion of vital organs with a stopped heart. While essential, CPB can induce the massive release of inflammatory cytokines, causing overwhelmed systemic inflammation reactions that may increase perioperative mortality.

It is estimated that the incidence of systemic inflammatory response syndrome (SIRS) in patients undergoing CPB can reach as high as 40% to 50% [2, 3]. During CPB, contact of blood with artificial materials activates monocytes and macrophages, both of which release inflammatory cytokines and induce inflammation [4]. In patients who developed SIRS after CPB, white blood cell count and multiple inflammatory markers such as interleukin (IL)-6, IL-8, and tumor necrosis factor-α (TNF-α) were all significantly increased [5–7]. In addition, the severity of SIRS, marked by inflammatory cytokines, after cardiac surgery directly correlates to the perioperative complications and prognosis [8–10].

Acute type A aortic dissection (ATAAD) is a cardiovascular emergency that requires immediate open surgical treatment. In the surgical treatment of ATAAD, the extensive repair necessitates prolonged use of CPB, which exacerbates inflammation and negatively impact surgical outcomes. Because the levels of inflammatory markers influence the severity of SIRS and prognosis, measures to reduce inflammation have always been the center of focus in open-heart surgery.

Hemoperfusion is a technique widely used in toxicology for the removal of toxins. It works by removing cytokines that cannot be filtered using existing blood purification techniques [11]. Recent studies suggested that hemoperfusion is also able to absorb inflammatory mediators produced during CPB and produce a long-lasting anti-inflammatory effect [12]. However, the role of hemoperfusion in the settings of ATAAD patients undergoing CPB and deep hypothermic circulatory arrest (DHCA) is currently unknown. In the present study, we would like to determine if hemoperfusion is able to reduce inflammatory mediators and reduce the perioperative complications in ATAAD patients undergoing CPB and DHCA.

### Objective and hypothesis

This study is a single-center, randomized, controlled, double-blind clinical trial. Subjects undergoing DHCA/CPB will be allocated to the hemoperfusion group and control group. The main purpose of this study is to determine if the levels of pro-inflammatory cytokines and anti-inflammatory cytokines differ between the two study groups in the perioperative period. In addition, differences in perioperative coagulation parameters, blood product usages in the intensive care unit (ICU), length of hospital stay, postoperative complications, and all-cause mortality between two groups will also be determined. We hypothesize that hemoperfusion could improve the overall prognosis in patients with acute type A aortic dissection undergoing cardiopulmonary bypass and DHCA.

## Methods

### Ethics and registration

**Fig. 1:**
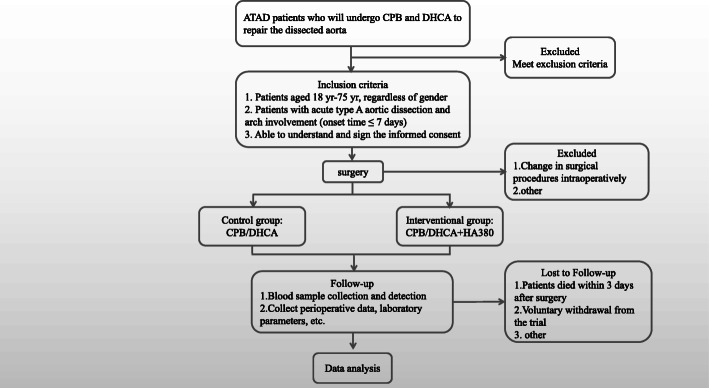
Flow chart of the hemoperfusion in acute type A aortic dissection patients undergoing aortic arch operation (HPAO) trial

This prospective, randomized, double-blind controlled study started recruiting patients at the Cardiovascular Center of the Second Affiliated Hospital of Nanjing Medical University in China in January 2019 and is expected to complete within 24 months (30 December 2020). The design of the trial follows the guidelines in the Helsinki Declaration and is in accordance with the guidelines of the Medical Research Involving Human Subjects Act and Good Clinical Practice (GCP). This trial has been approved by the Ethics Committee of the Second Affiliated Hospital of Nanjing Medical University (No.[2018] KY 118) and has been registered at ClinicalTrials.gov (NCT 04007484). According to the Consolidated Standards of Reporting Trials (CONSORT) statement, the study flow chart is developed, as shown in Fig. 1.

### Patient and public involvement

Patients or the public are not involved in the design, conduct, reporting, or dissemination plans of our research.

### Research objects

#### Inclusion criteria

1. Patients aged 18–75 years, regardless of gender
2. Patients with acute type A aortic dissection and arch involvement (onset time ≤ 7 days)
3. Able to understand and sign the informed consent

#### Exclusion criteria

1. Unable to understand and sign the informed consent
2. BMI ≥ 40
3. Pregnant
4. Active hemorrhage or thrombocytopenic purpura
5. Previous history of liver diseases, renal insufficiency, or cerebrovascular diseases
6. Prolonged operation time due to intraoperative accident
7. Preoperative organ malperfusion
8. Previous history of cardiac surgeries
9. Oral anticoagulant or antiplatelet drugs within 1 week of disease onset
10. Hereditary connective tissue diseases such as Marfan syndrome, Ehlers-Danlos, and Loeys-Dietz syndrome

The listed exclusion criteria are set to minimize the impact of perioperative variables, which may potentially undermine the validity of the study. The exclusion criteria are also backed up by a number of studies focusing on cardiac surgery [13–15].

### Experimental intervention/treatment

**Fig. 2:**
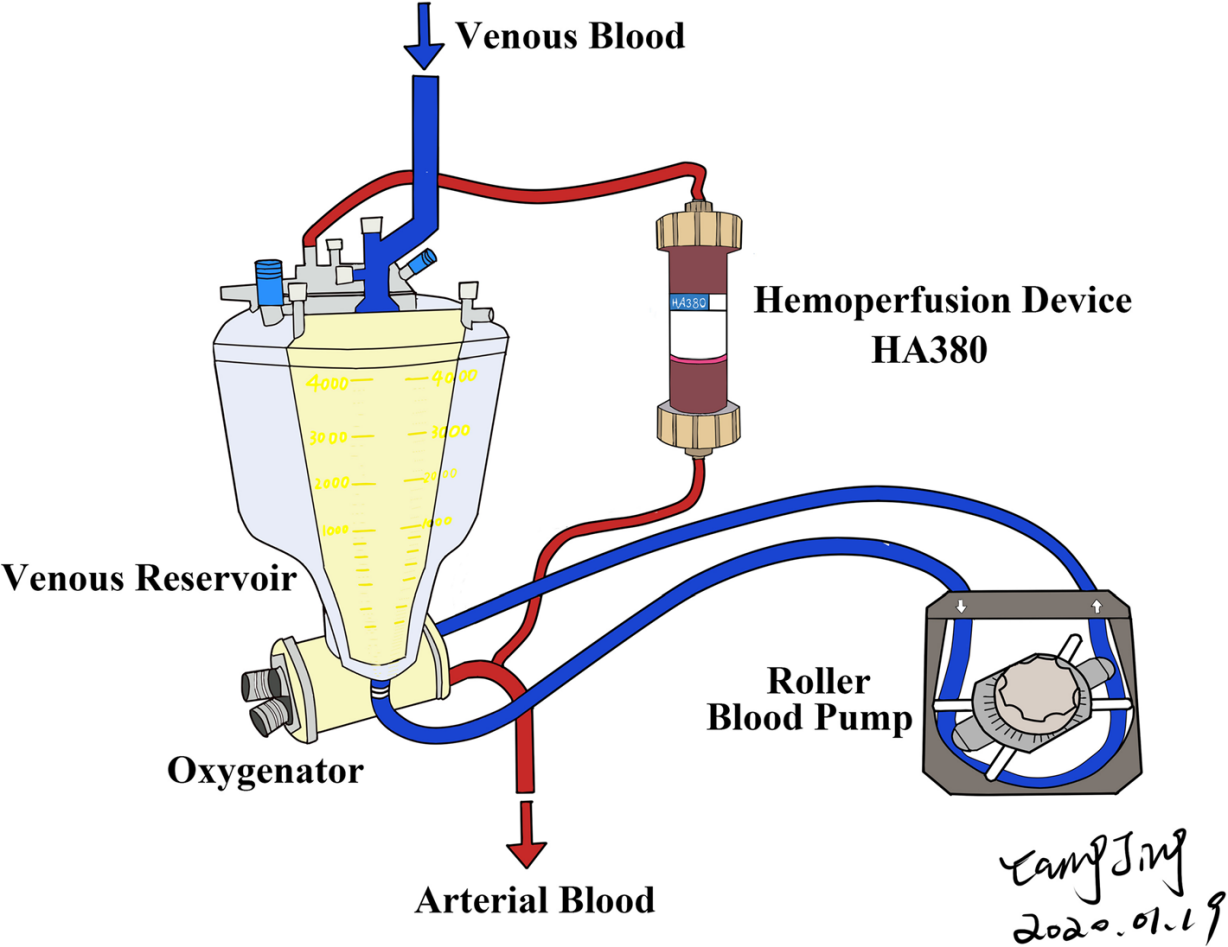
HA380 hemoperfutor setup in the CPB circuit. Venous blood first enters the venous reservoir, where it subsequently flows into the oxygenator with a driving force from a roller pump. After oxygenation, the majority of the oxygenated blood goes into the arterial circulation for perfusion, roughly 700 ml/min (14–18%) goes through the hemoperfusion device HA380 for hemofiltration. The filtered blood then returns to the venous reservoir and mixes with the venous blood. No other additional equipment is required

Hemoperfusion is achieved through blood filtration with a hemoperfusion device named HA380 (Zhuhai Jafron Biomedical, China). The hemoperfusion device, composed of multiple parallel macroporous adsorption resin cartridges, is connected in parallel to the oxygenator and blood reservoir and perfused at a rate of 400–700 ml/min (Fig. 2). Thanks to the large medium and macroporous structure and high specific surface area, HA380 is able to adsorb superfluous inflammatory factors and excess metabolites in the blood through different forces. The outbreak of inflammatory factors is thus expected to be controlled. The aim of hemoperfusion is to decrease inflammatory mediators produced during cardiopulmonary bypass through adsorption. The ultimate goal of hemoperfusion is to decrease postoperative complications, improve postoperative recovery, and shorten the length of hospital stay. Montin et al. detailed the safety and effectiveness of HA380. General recommendations of HA380 in cardiac surgery have also been made [16].

### Randomization and blinding

On the day of surgery, the perfusionists will explain to the patient’s families the context and significance of our trials and obtain informed consents. All informed consents will be kept in a separate folder and preserved by the project leader. Upon anesthesia, a statistician who does not participate in the experiments will use a computer software to randomly designate a number of either 0 or 1 to the patient, with 0 for the control group and 1 for the intervention group. The expected number of patients in the control group will equal that of the intervention group. To keep the trial confidential, the results of random allocation will only be known to the statisticians and perfusionists. Other physicians including cardiac surgeons, anesthesiologists, intensivists, and other clinicians will be blinded to the grouping. The details of randomization will not be accessible to anyone but the statisticians and perfusionists until the end of the trial and completion of the data analysis. If exclusion criteria are met after randomization (e.g., hemi-arch replacement is performed or deep hypothermia circulatory arrest not required), the patient will be removed from the trial.

During the hospital stay, only the perfusionists are aware of the grouping of patients. First of all, the size of HA380 is small. It is often not noticeable to the surgical team. Secondly, the shape of the device is no different to conventional filters used in CPB. Therefore, it is difficult to tell if HA380 is used just by eyeballing on the CPB circuit. Third, our HA380 are specially ordered and no label is visible on the device. Thus, only the perfusionist knows if the device was used. Cardiac surgeons, intensivists, and other clinicians actively involved in the treatment of the patients are blinded to patient grouping. The detail of randomization will not be revealed until the end of the study and completion of data analysis.

### Data collection using case report form (CRF)

Preoperative data including preoperative demographics (sex, age, BMI), details of presentation (onset time, symptoms such as chest pain, back pain, dyspnea, abdominal pain/abdominal distension, nausea/vomiting, coma, etc.), prior medical history (hypertension, hyperlipidemia, diabetes, coronary heart disease, kidney disease, cerebrovascular disease, smoking history, drinking history, etc.), preoperative imaging and laboratory results (such as electrocardiogram, cardiac ultrasound, computed tomography angiography, coagulation routine, liver and kidney functions, blood routine, etc.), and preoperative medications (norepinephrine, dopamine, urapidil, epinephrine, etc.) will be collected.

Intraoperative data including surgical methods, CPB details (CPB time, aortic cross-clamp time, circulatory arrest time, unilateral cerebral perfusion time, nasopharyngeal minimum temperature, etc.), volume of intraoperative blood products (such as suspended red blood cells, fresh frozen plasma, platelet, cryoprecipitate, etc.), amount of blood loss, and urine output will also be collected.

**Fig. 3:**
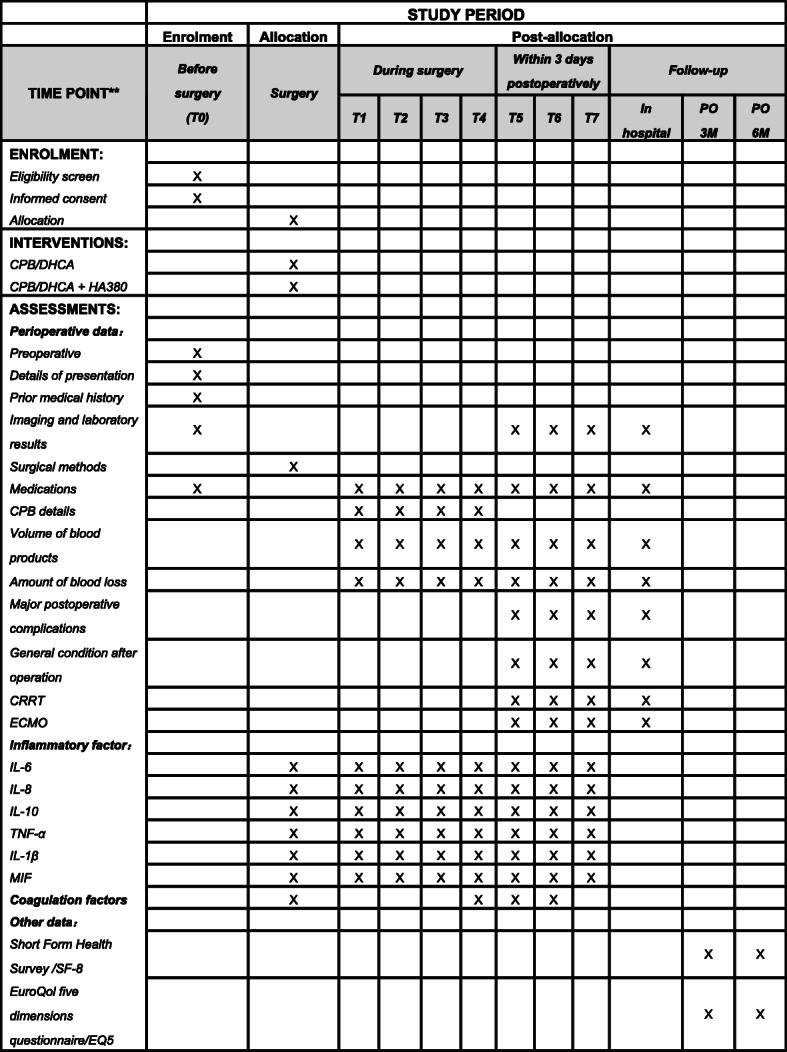
Schedule of enrollment, intervention, perioperative data, and laboratory parameters at different time points on the basis of Standard Protocol Items: Recommendations for Interventional Trials (SPIRIT) Statement. T0, before operation; T1, CPB initiation; T2, lowest intraoperative temperature; T3, end of CPB; T4, end of the operation; T5, 24 h after completion of the procedure; T6, 48 h after completion of the procedure; T7, 72 h after completion of the procedure; CPB, cardiopulmonary bypass; DHCA, deep hypothermic circulatory arrest; CRRT, continuous renal replacement therapy; ECMO, extracorporeal membrane oxygenation; MIF, macrophage migration inhibitory factor; PO 3M, postoperative 3 months; PO 6M, postoperative 6 months

Postoperative follow-up data including major postoperative complications (new renal failure-requiring dialysis, secondary thoracotomy, operative mortality, stroke/cerebrovascular accident, paraplegia, acute renal injury, respiratory failure, delirium, severe liver dysfunction, myocardial infarction, etc.), general condition after operation (duration of mechanical ventilation, drainage volume within 24 h postoperatively, urine volume within 24 h postoperatively, the length of ICU stay, all-cause mortality, dosage of vasoactive drugs, total hospitalization cost, etc.), and volume of postoperative blood product transfusions will be collected. Utilization of invasive postoperative treatment methods such as continuous renal replacement therapy (CRRT) or extracorporeal membrane oxygenation (ECMO) will be documented as well. In general, all patients enrolled will be followed up at our center. Part of the data to be collected throughout the study is shown in Fig. 3.

It should be noted that contrast-enhanced thoracic computed tomography aortography (CTA) needs to be provided before operation to confirm the diagnosis, and the data of preoperative examinations is the closest one to the operation. Preoperative and postoperative data will be collected and recorded by team members who are blinded to surgical details and randomization. Intraoperative data will be collected by perfusionists and anesthesiologists.

### Anesthesia and surgical procedures

Venous access is achieved through peripheral vein puncture in the operating room. Throughout the surgery, five-lead electrocardiogram (ECG), blood oxygen saturation (SPO_2_), and heart rate (HR) should be routinely monitored with patient monitor (Philips, Holland). Non-invasive cerebral oxygen saturation (rSO_2_) and BIS monitoring are achieved with Disposable sensor (Covidien, USA). During the operation, the value of rSO_2_ is maintained at no less than 80% of baseline with an absolute value greater than 50%. Induction of anesthesia is achieved with etomidate 10 to 15 mg, sufentanil 0.5–1 μg/kg, and cis-atracurium 0.15 mg/kg intravenously. Anesthesia is maintained with dexmedetomidine 0.2–0.4 μg/kg/h, sufentanil 1–3 μg/kg/h, cis-atracurium 0.06–0.12 mg/kg/h, and sevoflurane, at a BIS between 40 and 60.

After median thoracotomy, the supra-aortic branches are isolated. Once ACT reaches 480 s, cardiopulmonary bypass is started. Throughout the CPB, the flow rate is kept at 2 to 2.5 l/min/m^2^, and the mean arterial pressure is maintained between 50 and 70 mmHg (1 mmHg = 0.133 kPa). Cooling down of the patient is subsequently initiated while ascending aorta is being opened and false lumen thrombus being removed. Subsequently, the dissected proximal aorta is replaced with or without replacement of the prosthetic aortic valve while the patient is being cooled down. To minimize cerebral ischemia, arch vessels are reconstructed with the following sequence respectively: the left carotid artery, the left subclavian artery, and the right innominate artery. After completion of repair and rewarming, the patient is weaned from CPB. Protamine is given to reverse the effect of heparin if needed. The procedure completes after hemostasis is reached and the sternum is closed.

### Blood sample collection and detection

During the perioperative period, plasma inflammatory factors levels will be measured at the following time points: before operation (T0), CPB initiation (T1), lowest intraoperative temperature (T2), end of CPB (T3), end of the operation (T4), 24 h after completion of the procedure (T5), 48 h after completion of the procedure (T6), and 72 h after completion of the procedure (T7).

Parameters of coagulation including plasma-activated partial thromboplastin time (APTT), prothrombin time (PT), fibrinogen (FIB), fibrinogen degradation product (FDP), and d-dimer (DDi) levels will be measured at the following time points: before operation, 24 h after completion of the procedure (POD 1), 48 h after completion of the procedure (POD 2), and 72 h after completion of the procedure (POD 3).

Similarly, levels of coagulation factors including II, VII, IX, X, XI, and XII will be measured at the following time points: before operation, end of CPB, end of the operation, 24 h after completion of the procedure (POD 1), and 48 h after completion of the procedure (POD 2).

Measurement of inflammatory cytokines levels: blood samples are collected through radial artery catheterization. The first 5 ml will be discarded when sampling. Following collection, blood samples are immediately centrifuged at 3000 rpm for 15 min at 4 °C. The plasma yielded will be stored in the − 80 °C refrigerator till the time of measurements. Enzyme-linked immunosorbent assay (ELISA) is used for the measurement of inflammatory cytokines.

### Primary outcome measures

1. Incidence of composite major complications [time frame: up to 30 days]:
  Operative mortality
  Secondary thoracotomy
  New onset of postoperative renal failure requiring dialysis: increase in serum creatinine value ≥ 300% from baseline or an absolute serum creatinine ≥ 354 μmol/l with an increase ≥ 44 μmol/l from baseline (KDIGO stage 3) [17]. Any patient who received acute dialysis is put into this category. Patients who have received dialysis within 1 month prior to the surgery are not eligible for this endpoint
  Paraplegia
  Stroke/cerebrovascular accidents
  Low cardiac output syndrome: cardiac index < 2.0 l min^−1^ m^−2^ for > 8 h after surgery, regardless of treatment [18]
  Reintubation
  Severe liver dysfunction: ALT level above 500 U/l or a total bilirubin above 3.0 mg/dl [19, 20]
  ECMO support
  Multiple organ dysfunction syndrome (MODS)

Each of these outcomes will be analyzed as a secondary endpoint.

### Secondary outcome measures

1. Changes in the levels of plasma inflammatory factors such as IL-6, IL-8, IL-10, IL-1β, and TNF-α [time frame: up to 3 days]
2. Changes of plasma MIF levels during the perioperative period [time frame: up to 3 days]
3. Changes of plasma CRP levels during the perioperative period [time frame: up to 3 days]
4. Changes of the plasma II, VII, IX, X, XI, and XII levels during the perioperative period [time frame: 2 days]
5. Changes of the plasma APTT, PT, FIB, FDP, and DDi levels during the perioperative period [time frame: 3 days]
6. Total drainage within the first 24 h of surgery [time frame: 24 h]
7. Incidence of postoperative acute kidney injury [time frame: up to 30 days]: increase in serum creatinine ≥ 150% or ≥ 26.5 μmol/l from baseline [13]
8. Incidence of postoperative respiratory failure [time frame: up to 30 days]: uninterrupted postoperative mechanical ventilation for more than 48 h [10]
9. Incidence of postoperative delirium [time frame: up to 30 days]: we will assess delirium once a day for a total of 3 times during the first 72 h after ICU admission, using standard delirium assessments (CAM or CAM-ICU). Before the CAM-ICU will be performed, a Richmond Agitation Sedation Scale (RASS) sore should be evaluated in advance [21]
10. Incidence of postoperative liver injury [time frame: up to 30 days]: ALT level above 150 U/l [22]
11. Incidence of postoperative myocardial infarction [time frame: up to 30 days]: a rise and/or fall of cTn values with at least 1 value above the 99th percentile upper reference limit and at least 1 of the following:
  Symptoms of myocardial ischemia
  New ischemic ECG changes
  Development of pathological Q waves
  Imaging evidence of new loss of viable myocardium or new regional wall motion abnormality in a pattern consistent with an ischemic etiology
  Identification of a coronary thrombus by angiography or autopsy (not for types 2 or 3 MIs) [23]

### Other outcome measures

1. The volume of blood transfusion within ICU [time frame: up to 30 days]
2. The need of vasoactive drugs [time frame: up to 30 days]
3. Length of ICU stay [time frame: up to 30 days]
4. Length of postoperative hospital stay [time frame: up to 30 days]
5. Prolonged postoperative extubation (> 48 h) [time frame: up to 30 days]
6. Total hospital expenses [time frame: up to 6 months]

### Sample size calculation

Primarily, we expect the incidence of composite major complications to be decreased in the HA380 hemoperfusion group (intervention group) compared with the control group. The sample size was calculated with our preliminary results from the pilot study, in which the primary outcome was defined as the incidence of composite major complications. In this study, we included 24 patients (*n* = 12 in each group), in which 8.3% (1 out of 12) of the patients assigned to the intervention group underwent CRRT due to severe postoperative renal failure. In contrast, 33.3% (4 out of 12) patients had either one of the following outcomes: death, secondary thoracotomy, and renal failure requiring dialysis. Assuming a two-sided type I error probability (α) of 5% and type II error probability (β) of 20%, forty patients are needed in each group. Suppose 10% of patients drop out either due to changes in surgical methods or lost in follow-up, a minimum number of 44 patients in each group are needed in order to achieve statistical significance.

### Data management and quality control

A safety monitoring committee composed of cardiovascular surgeons, anesthesiologists, perfusionists, and statisticians will be set up to monitor the progress and safety of the clinical study, including adverse events and mortality. All adverse events will be recorded in the case report form with severity assessed. A team member who is not involved in grouping, surgery, and postoperative management will be assigned to summarize the data. If for any reason a subject could not adhere to the study, he or she will be removed from the study groups and the sampled data will also be abandoned. On rare occasions, some of the blood samples withdrawn before emergent surgery may not meet the requirement for lab studies (hemolysis happened during blood withdrawn, blood clotted or the sample was contaminated, etc.). Should it occur, multiple imputation will be used to handle the missing data. However, if many pieces of data are missing, or vital data are missing (such as patient outcomes), the subject will be removed from the study group. Throughout the trial, only the project leader has access to the patient’s data and make the final decision to terminate the trial. All information will be shared with the trial team upon completion of the study.

For quality control, all perfusionists and anesthesiologists will receive unitive training to ensure correct installation and operation of the HA380 hemoperfutor throughout the trial. The trial is overseen by the Ethics Committee of the 2nd affiliated hospital of Nanjing Medical University and will be audited every 6 months. All saved data will be kept confidentially in accordance with standard guidelines. All changes to the data first keyed in will be documented and a valid reason is required for data revision.

### Data analysis

After completion of the trial, data analysis will be done by a certified statistician with SPSS20.0 (IBM, Chicago, IL, USA). Levels of inflammatory cytokines and coagulation parameters will be expressed as mean ± standard deviation or median (interquartile range). An independent *t* test or the Wilcoxon signed-rank test will be used to check if the data is within normal distribution before analysis. Categorical variables will be analyzed with chi-squared and Fisher’s exact tests.

The relative risk of primary and secondary outcomes in the hemoperfusion group and the control group will be compared using the multivariate logistic regression. Kaplan-Meier curves and log-rank analysis will be used to compare inter-group differences in primary and secondary outcomes. When comparing the changes in coagulation factor levels during the perioperative period, the effect of hemoperfusion on coagulation factors throughout the study will be assessed using repeated measures’ analysis of variance (ANOVA) and analysis of covariance (ANCOVA). When the *p* < 0.05, the result is considered statistically significant.

## Discussion

During CPB, increased levels of pro-inflammatory cytokines and decreased levels of anti-inflammatory cytokines activate the immune system [24, 25]. The inflammation can be even more severe when deep hypothermic circulatory arrest is needed.

Most cytokines are small soluble proteins (< 40 kDa) involved in the signal transduction pathways that play key roles in the immune response [26]. The balance of pro-inflammatory and anti-inflammatory cytokines often determines the pattern of inflammation. When there is an uncontrolled release of pro-inflammatory cytokines and overactivation of the immune system, a hyperinflammatory state called cytokine storms ensues. This significantly complicates the postoperative recovery and increases the incidence of MODS and death [27–29]. We therefore set the incidence of composite major complications as the primary outcome in the instant study.

Major proinflammatory cytokines produced in CPB include IL-1, IL-6, IL-12, and TNF-α [24]. IL-6, a cytokine released by tissue macrophages, is a pro-inflammatory cytokine that is increased early during the inflammatory process. Its level increases significantly after initiation of CPB and peaks at 2–4 h after the end of CPB [7]. Additionally, increased levels of IL-6 increase the incidence of postoperative complications such as ARDS, myocardial ischemia, and low cardiac output syndrome [6]. Measures in decreasing the amount of IL-6 has been suggested to preserve vascular barrier integrity, reduce capillary leakage, and alleviate postoperative inflammations [30]. Consequently, we included the levels of IL-6 as one of the secondary outcomes.

Anti-inflammatory cytokines (such as IL-1RA, IL-4, and IL-10) play an equally important role in host defense [31, 32]. Among the aforementioned cytokines, IL-10 is an important anti-inflammatory cytokine that regulates the compensatory anti-inflammatory response syndrome (CARS) against SIRS [33]. However, elevated IL-10 levels can also increase the incidence of postoperative infection and sepsis [34]. Therefore, assessing the levels of IL-10 can help predict the incidence of postoperative infections.

Our study will also aim to evaluate the changes of postoperative blood coagulation function and determine the new mechanisms of postoperative organ failure. Macrophage migration inhibitory factor (MIF) is a secretory homotrimer that plays a key role in promoting the production of proinflammatory cytokines [35–37]. With a molecular weight of 12.5 kD, MIF can be effectively removed by the HA380 hemoperfusion. Removal of MIF may have a protective effect on the central nervous system and reduce the incidence of postoperative delirium. Further, free hemoglobin and myoglobin can also be readily removed by the HA380 hemoperfutor during CPB. Because both hemoglobin and myoglobin are involved in the occurrence of cardiac surgery-associated acute kidney injury (CSA-AKI) [38], hemoperfusion may have an additional protective effect on the kidney and may contribute to the recovery of renal function after cardiac surgery.

Because the interplay between inflammation and coagulation systems seriously worsens the prognosis of patients undergoing surgery, we therefore aim to evaluate if hemoperfusion could reduce abnormal bleeding and the amount of transfusion after the aortic arch operation. Additionally, in order to evaluate the safety of HA380 hemoperfutor, we will also detect the levels of perioperative coagulation factors such as II, VII, IX, X, XI, XII, and so on.

Steroids are commonly used for the excessive inflammation in ATAAD patients with severe postoperative inflammation [39]. While effective, steroid uses are not without side effects. Hyperglycemia and increased risk of postoperative infection are common side effects seen with steroids. Another anti-inflammatory drug, ulinastatin, can cause granulocytopenia and allergies. Therefore, other emerging technologies such as hemofiltration are needed to address the limitations of medications. Though well tolerated, one limitation with the hemoperfusion technologies is the inconsistent filtration and adsorption results.

HA380 is a new hemoperfusion device [16]. In this trial, we evaluated the effect of the hemoperfusion device (HA380) on the perioperative inflammatory responses in ATAAD patients undergoing CPB and DHCA. The purpose of this study is to investigate if hemoperfusion with HA380 is sufficient to reduce perioperative proinflammatory cytokine levels and reduce the incidence of major postoperative complications. Additionally, we also explored the effect of HA380 hemoperfusion on coagulation, hemodynamics, and clinical outcomes. Upon completion of the study, we will elucidate the mechanism of hemoperfusion on SIRS. Further, this study helps to provide evidence for the application of HA380 in ATAAD patients undergoing CPB and DHCA.

### Strengths and limitations of this study

- ➢ This is the first randomized controlled trial (RCT) to evalutae the effect of combined HA380 hemoperfusion and CPB/DHCA versus conventional CPB/DHCA in patients with ATAAD undergoing procedures involving the aortic arch.
- ➢ Interventions during CPB/DHCA aims to decrease the levels of pro-inflammatory cytokines in the perioperative period and reduce severe postoperative complications.
- ➢ This is a double-blind clinical trial. While it is not possible to blind the perfusionists from the patient’s grouping, bias will be minimized by assigning other independent personnel for data collection at different clinical stages.
- ➢ In the future, a large randomized controlled trial is needed, given the results of this single-center trial.

### Trial status

The protocol was submitted to and approved by the Ethics Committee of the Second Affiliated Hospital of Nanjing Medical University (No.[2018] KY 118) on 20 December 2018. Patient recruitment started on 2 January 2019 and is expected to complete by 30 December 2020 (24 months). Following retrospective registration at ClinicalTrial.gov, the protocol was first released on 1 July 2019. The final protocol version is version 4, 29 February 2020.

## Data Availability

Not applicable.

## Abbreviations

CPB: Cardiopulmonary bypass
SIRS: Significant systemic inflammatory response syndrome
ATAAD: Acute type A aortic dissection
DHCA: Deep hypothermic circulatory arrest
IL: Interleukin
TNF-α: Tumor necrosis factor-α
ICU: Intensive care unit
GCP: Good Clinical Practice
CONSORT: Consolidated Standards of Reporting Trials
CRF: Case report form
CTA: Computed tomography angiography
CRRT: Continuous renal replacement therapy
ECMO: Extracorporeal membrane oxygenation
ECG: Electrocardiogram
SPO_2_: Blood oxygen saturation
HR: Heart rate
rSO_2_: Regional cerebral oxygen saturation
ACT: Activated coagulation time
PO 3 M: Postoperative 3 month
PO 6 M: Postoperative 6 month
POD: Postoperative day
APTT: Activated partial thromboplastin time
PT: Prothrombin time
FIB: Fibrinogen
FDP: Fibrinogen degradation product
DDi: D-Dimer
ELISA: Enzyme-linked immunosorbent assay
MODS: Multiple organ dysfunction syndrome
MIF: Macrophage migration inhibitory factor
CRP: C-reactive protein
SPIRIT: Standard Protocol Items: Recommendations for Interventional Trials
ANOVA: Analysis of variance
ANCOVA: Analysis of covariance
CARS: Compensatory anti-inflammatory response syndrome
CSA-AKI: Cardiac surgery-associated acute kidney injury
RCT: Randomized controlled trial
